# Morphometric analysis of the intergenerational effects of protein restriction on nephron endowment in mice

**DOI:** 10.1016/j.heliyon.2024.e39552

**Published:** 2024-10-18

**Authors:** Fabiola Diniz, Francesca Edgington-Giordano, Nguyen Yen Nhi Ngo, Gal Caspi, Samir S. El-Dahr, Giovane G. Tortelote

**Affiliations:** Section of Pediatric Nephrology, Department of Pediatrics, Tulane University School of Medicine, New Orleans, LA, 70112, USA

**Keywords:** Kidney, Low protein diet, Developmental programming, Nephron endowment, Hypertension, Oligonephropathy

## Abstract

**Background:**

Parental nutritional status is crucial in shaping offspring's kidney development. However, the association between a protein-restrictive diet and its intergenerational impact on kidney development remains unclear.

**Methods:**

We conducted multigenerational morphometric measurements to investigate the effects of parental protein deprivation on offspring kidney development across four generations. F0 mice were divided into two groups and fed a normal protein diet (NPD) or a low-protein diet (LPD) for three weeks before mating and continued these diets throughout gestation and lactation. Body weight (BW), kidney weight (KW), KW/BW ratio, nephron counts, and blood pressure were assessed in F1 pups. To examine paternal effects, we bred CD1 females on an NPD with males on an LPD. BW, KW, KW/BW, and nephron counts were measured at P20. To measure the transgenerational effect of parental LPD on kidney development, F1 offspring (from parents on LPD) were fed NPD upon weaning. These F1 offspring were bred at 6 weeks of age to produce F2, F3 and F4 generations. Kidney metrics were evaluated across generations.

**Results:**

The average body weight of P0 pups from parents on NPD was 1.61g, while pups from parental LPD weighed an average of 0.869g, a decrease of 54 % (p = 6.9e-11, Wilcoxon test). F1 from parental LPD have significantly smaller kidneys than the control, with an average combined kidney weight of 0.0082g versus 0.0129g, a 37 % decrease (p = 3.2e-02, Wilcoxon test). P20 BW and KW remained low in LPD offspring. These effects persisted for 4 generations (F1 to F4) with an average glomerular count reduction of roughly 20 %. F3 and F4 showed wider variability in glomerular counts but were not statistically significant compared to controls.

**Conclusions:**

Both maternal and paternal LPD significantly affected offspring nephron endowment. Our study underscores the complex nature of nutritional transgenerational effects on kidney development, emphasizing the importance of both maternal and paternal dietary impacts on kidney development and the developmental origin of adult disease.

## 1 Introduction

Parental deficient nutrition, especially during pregnancy, causes long-term changes in fetal gene expression, altering fetal metabolism and leading to Renal-metabolic-cardiovascular abnormalities later in life [[1], [2], [3], [4], [5], [6], [7], [8]]. Epidemiological studies have shown correlations between lower birth weight and increased risk of adult hypertension, coronary heart disease, and impaired glucose tolerance or insulin resistance [6,7,[9], [10], [11], [12], [13]]. Notably, birth weight indicates intrauterine environment quality, and in itself, it is not the primary cause of health problems. Lower birth weight results from a suboptimal intrauterine environment, which in turn is correlated to oligonephropathy at birth. This condition is a significant risk factor for the development of hypertension and chronic kidney disease later in life [9,12,[14], [15], [16], [17]].

Extensive evidence has supported the hypothesis that an adverse uterine environment during the prenatal period predisposed offspring to hypertension, and chronic kidney diseases (CKDs) [9,[11], [12], [13], [14], [15],^18^]. Barker's pioneering work in the late 1980s and early 1990s showed that low birth weight was associated with increased rates of heart disease in adulthood. Later Brenner's contributions further elucidated the mechanisms by which early life conditions influence kidney development and subsequent disease risk [9,11,14,[18], [19], [20]]. Their research indicated that inadequate nutrition during the periconceptional and prenatal periods increases the likelihood of these non-communicable diseases in offspring.

Both human and rodent studies indicate that the kidney, especially the number of nephrons, plays a crucial role in the development of hypertension [14,[18], [19], [20], [21], [22]]. Surprisingly, there is limited data on the impact of parental low-protein diet (LPD) on kidney development in subsequent generations, especially in mice. We and others have demonstrated that when parents (hereafter referred to as the F0 generation) are given an LPD, the normal kidney development of the offspring (referred to as the F1 generation) is altered, potentially leading to a lifelong increase in blood [3,5,[23], [24], [25], [26]].

Mounting evidence indicates that, beyond intrauterine nutritional stress, gestational malnutrition can impact epigenetic regulation [[27], [28], [29], [30], [31], [32], [33]]. This raises the possibility that periods of undernutrition can create heritable changes to the epigenome, meaning the effects of undernutrition during the fetal period might not be limited to the first generation. Previous observations with humans and rodents suggest that transgenerational effects can be passed on to future generations through epigenetic mechanisms [16,17,31,[34], [35], [36]].

In a recent study using single-cell RNA sequencing, we found that fetal exposure to an LPD reduced nephron numbers by approximately 25 % at birth. The underlying molecular mechanism involved alterations in the transcription of genes related to cell proliferation, differentiation, and metabolism, which disrupted the developmental path of kidney progenitor cells. These changes ultimately resulted in oligonephropathy at birth [5]. However, it remains unclear whether these effects are limited to the offspring or if they would impact future generations.

In this study, we investigated the direct and transgenerational effects of parental protein restriction on nephron endowment across four generations.

## 2 Methods

**Ethical Considerations:** All animal procedures were approved by the Institutional Animal Care and Use Committee (IACUC), protocol #1558, and conducted in accordance with the National Institutes of Health guidelines for the care and use of laboratory animals.

**Diet:** Food and water were provided ad libitum. The protein-restricted experimental diet used for this study was acquired from Envigo (TD 90016). This LPD is isocaloric and has 6.5 % protein, 80.4 % carbohydrate, 13.1 % fat, and 3.8 kcal/g. The control diet is Envigo TD 91352 has 21.6 % protein, 65.4 % carbohydrate, 13.0 % fat, and 3.8 kcal/g. The only difference between the two diets is the amount of protein, the 6 % protein diet has more sucrose and cellulose to maintain an iso-caloric formula (Table 1).

**Table 1:** Diet composition. Note the significant differences in protein content.

Ingredient	20 % protein Diet (g/Kg)	6 % Protein Diet (g/Kg)
**Casein**	230	69
**DL-Methionine**	3	0.9
**Sucrose**	431.7	571.8
**Corn Starch**	200	200
**Corn Oil**	52.3	53.9
**Cellulose**	37.86	57.82
**Vitamins Mix, Teklad 40060**	10	10
**Ethoxyquin, antioxidant**	0.01	0.01
**Mineral Mix**	13.37	13.37
**Calcium Phosphate dibasic**	16.66	21.6
**Calcium Carbonate**	5.1	1.6

**Nutrient**	**Control Diet %** **kCal From**	**6 % Protein Diet %** **kCal From**

**Protein**	21.6	6.5
**Carbohydrate**	65.4	80.4
**Fat**	13	13.1

**Mouse Model and Breeding:** CD1 female and male mice were acquired from the Charles River laboratory and bred in-house for this study. Male and female mice were put on a specific diet (NPD or LPD) for 3 weeks starting at weaning (P20) and before pairing. Diets were continued during all pairing, pregnancy, and lactation periods. At postnatal day zero (P0) all growing litters were culled to 10 pups each. This measure normalizes litter size and helps control postnatal food access.

**F1 to F3 generation from F0.** Twenty-one-day-old CD1 mice (male and female) were assigned to either an NPD or an LPD for three weeks. After this period, these mice were bred to produce the F1 generation. Females in the proestrus or estrus phase were mated with males overnight [37]. Pregnancy was confirmed by the presence of a vaginal plug the following morning. The breeding scheme comprised three original groups: 1) male and female on NPD; 2) male and females on LPD; and 3) males on LPD and females fed an NPD. These NPD and LPD offspring were either collected for analysis at different time points or placed on NPD at P20 and bred to generate an F2 generation. This procedure was repeated to produce an F3 and F4 generation. To evaluate the paternal effect of LPD on the offspring, Male LPD mice were bred to a female o NPD, and the offspring were collected and analyzed as described below.

**F1-F3 from LPD-fed F0 Characterization:** All mouse pups were weighed at birth. Kidney weight from harvested kidneys was measured after the removal of the ureter and capsule for combined kidney weight (left kidney + right kidney). At P20 mice were weighed and kidneys were harvested with capsule and ureter removed for combined kidney weight at P20. Animals kept for blood pressure measurements were maintained in the mouse colony and fed a regular chow.

**Systolic blood pressure measurement:** Tail-cuff plethysmography was used to measure blood pressure in awake mice from both groups (NPD and LPD). This is a non-invasive procedure with physical restraint limited to less than 30 min. The equipment was placed in the vivarium to minimize animal stress. Systolic blood pressure was monitored using an automated non-invasive tail-cuff plethysmography technique (Visitech system, Apex, NC). Animals were acclimated to the holder restraints for two days, and measurements were obtained over 3 consecutive days to reduce the impact of restraint-induced stress. Ten to 15 consecutive measurements were taken for each animal while warming at 35 degrees C under slight restraint.

**Glomerular Count:** Collected kidneys were fixed in 10 % formalin for 24 h at room temperature and embedded in paraffin blocks. Subsequently, these paraffin-embedded kidneys were sectioned at 5 μm, and hematoxylin and eosin (H&E) staining were used to visualize kidney structures. A large image grab was created to show a complete mid-coronal image of the kidney section obtained using a high-resolution Nikon microscopy model Eclipse Ni. Next, fully formed glomeruli were counted in sections that were 5 μm thick and 15 μm apart, (given that on average a mature glomerulus measures roughly 55–85 μm). We counted 3 mid-coronal sections that are 15 μm and averaged those counts to produce an average count per section for that kidney.

**Image capture system:** The images were captured with a Zyla5/Andor camera mounted on a Nikon Eclipse Ni fluorescent microscope. All images were processed with the Nikon NIS-Elements platform version 5.21.03. The final files were saved as TIFF with a resolution greater than 300 dpi.

**Sex-Specific Comparisons:** data were also analyzed separately for male and female mice to identify any sex-specific differences.

**Statistical Analysis:** Comparisons between NPD and LPD groups were conducted using the Wilcoxon test, with significance set at p < 0.05. The calculations were made in R programming language (4.3.3). the following libraries were used for statistical calculations and plotting: ggpubr [38], and Tydiverse [39]. No proprietary codes were generated.

## 3 Results

Twenty-one-day-old CD1 mice, both males and females, were assigned to either a Normal Protein Diet (NPD) or an LPD for three weeks before mating. After this period, these mice were bred to produce the F1 generation. The breeding scheme included three original groups: 1) males and females fed an NPD, 2) males fed LPD and females fed NPD and 3) males and females fed LPD.

The mating pairs from the NPD and LPD groups (F0 generation) were kept separate throughout the experiments. The F1 generation, consisting of NPD and LPD offspring, were either collected for analysis at P0 and P20 or placed on an NPD diet after weaning (P20). At approximately 5 weeks of age, these F1 mice were bred to produce the F2 generation.

**Fig. 1:**
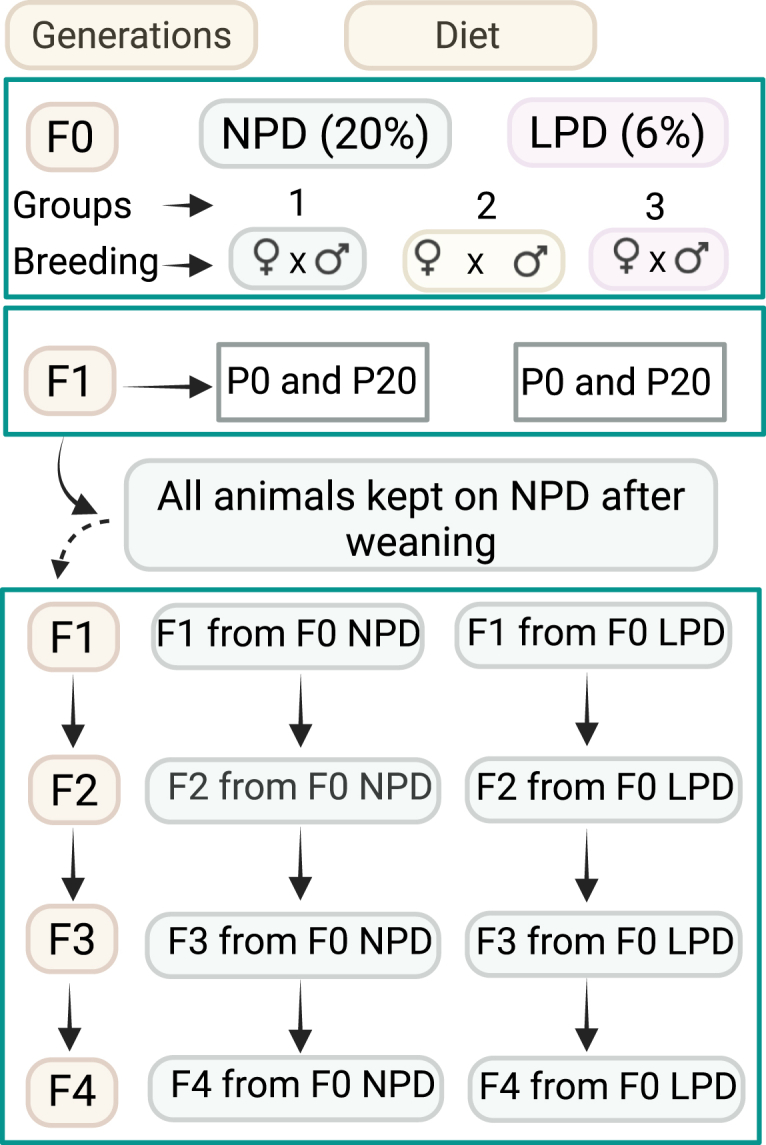
Study design. Twenty-one-day-old CD1 mice (both males and females) were assigned to either a Normal Protein Diet (NPD) or a Low Protein Diet (LPD) for three weeks. After this period, these mice were bred to produce the F1 generation. The breeding scheme comprised three original groups: 1) male and female on NPD; 2) males on LPD and females fed an NPD and 3) males and females on LPD. These NPD and LPD offspring were either collected for analysis at different time points (P0 and P20) or placed on NPD at weaning (P20) and bred to generate an F2 generation. This procedure was repeated to produce an F3 and F4 generation. The collected tissues were analyzed as described in the methods section. Parental generation and start point (F0); F1 – F4 subsequent generations. Notice that only the F0 generation was put on an NPD or LPD, all subsequent generations were fed an NPD.

The F2 generation was bred to produce the F3 generation, and the F3 generation was bred to produce the F4 generation (Fig. 1). It is important to note that only the F0 breeding pairs were exposed to the LPD. To ensure consistent litter size and food availability, only 10 newborn pups were kept per litter, corresponding to the 10 functional mammary glands in female mice (five on each side).

### 3.1 Parental LPD impact on mouse and kidney development at birth (P0)

Nephrogenesis in mice begins around mid-gestation, at embryonic day 10.5 (E10.5), and continues until about a week after birth [40]. To evaluate the impact of a low-protein diet on final nephron endowment, we compared nephron counts in F1 offspring from F0 parents on either an NPD or LPD.

**Fig. 2:**
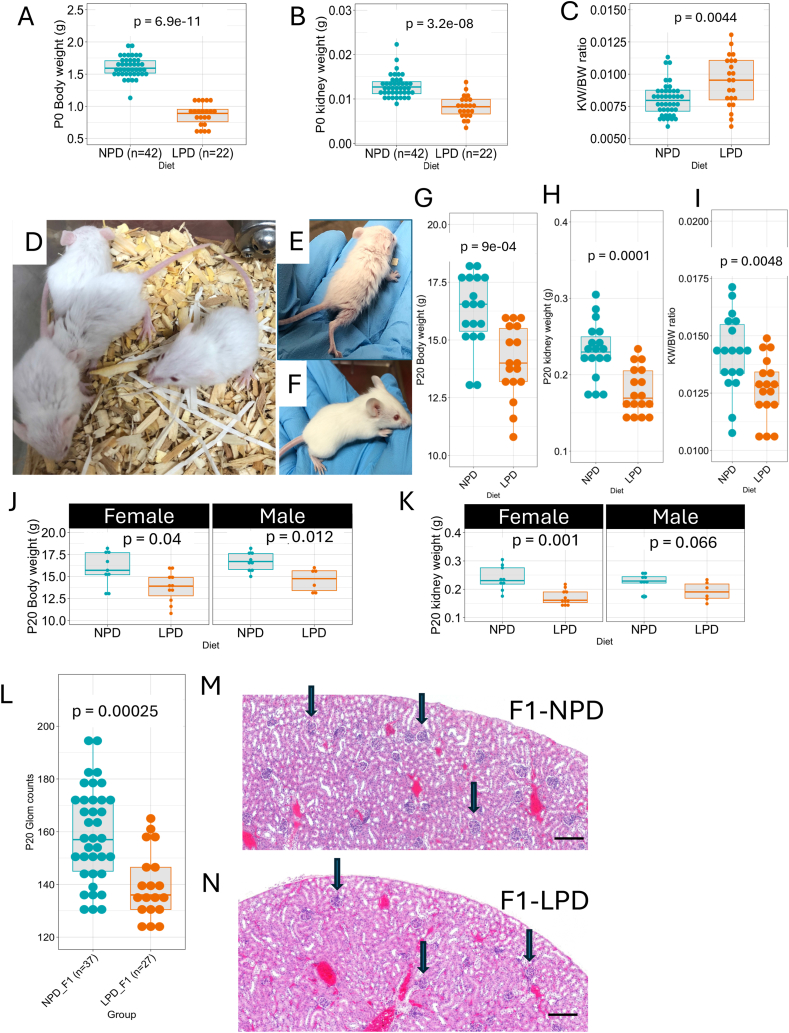
**LPD impact on mouse and kidney development.** P0 LPD offspring are born with lower body weight than control (BW mean ± sd NPD F1 = 1.61 ± 0.15g, n = 42; LPD F1 = 0.87 ± 0.17g, n = 22, p = 6.9e-11, Wilcoxon test) (A). LPD offspring mice have decreased kidney weight (KW mean ± sd NPD F1 = 0.013 ± 0.002g; LPD F1 = 0.008 ± 0.002g, p = 3.2e-8, Wilcoxon test) (B). The KW/BW ratio was increased in the LPD offspring KW/BW mean ± sd NPD F1 = 0.008 ± 0.001g; LPD F1 = 0.009 ± 0.001g, p = 0.004) (C). Morphological analysis of P20 F1 offspring shows the impact of LPD on normal development (D–F). P20 Body weight (NPD F1 = 16.3 ± 1.57g, n = 18; LPD F1 14.0 ± 1.57g, n = 17, p = 9e-4, Wilcoxon test) and kidney weight (NPD 0.232 ± 0.0369g; LPD 0.179 ± 0.0297g, p = 0.00012, Wilcoxon test) were significantly reduced in LPD offspring (G, H). The KW/BW ratio was decreased in the P20 LPD offspring (KW/BW NPD F1 = 0.0138 ± 0.00180g, 18; LPD F1 = 0.0127 ± 0.00133g, n = 17, p = 0.0048, Wilcoxon test) (I). LPD impacted body weight and kidney weight in both males and females to a similar extent (J and K). The glomerular counts at P20 were reduced in the F1 generation from F0 fed an LPD diet (NPD F1 = 158 ± 18, 37; LPD = 129 ± 21, 27, p = 0.00025, Wilcoxon test) (L). H&E staining of tissue sections from the P20 kidney of parental NPD and LPD groups, the round structures indicated with black arrows are glomeruli (M and N). Values are presented as mean ± sd. Scale bar 200 μm.

The initial impact of LPD on mouse and kidney development was strikingly evident at birth (P0) (Fig. 2). LPD offspring were born with significantly lower body weight compared to controls (BW NPD F1 = 1.61 ± 0.15g, n = 42; LPD F1 = 0.87 ± 0.17g, n = 22, p = 6.9e-11, Wilcoxon test) (Fig. 2A). Additionally, LPD offspring had reduced kidney weight (KW NPD F1 = 0.013 ± 0.002g; LPD F1 = 0.008 ± 0.002g, p = 3.2e-8, Wilcoxon test) (Fig. 2B). However, the KW/BW ratio was increased in LPD offspring (KW/BW NPD F1 = 0.008 ± 0.001g; LPD F1 = 0.009 ± 0.001g, p = 0.004) (Fig. 2C). Interestingly, the KW/BW ratio in control kidneys was, on average, 0.8 % of body weight, whereas in the F1 LPD group, it was 0.95 % of total body weight. Thus, although LPD F1 offspring have lower body and kidney weight their kidney/body weight ratios are higher than control pups.

These initial findings show that prenatal protein restriction has an immediate and substantial impact on fetal growth and kidney development (Fig. 2), confirming Barker and Brenner's hypothesis [[9], [10], [11]] and setting the stage for potential long-term health consequences.

### 3.2 Postnatal impacts of parental LPD on mouse and kidney development

LPD offspring mice exhibited noticeable morphological differences in growth and development compared to control mice. At birth, LPD offspring were thin, with bright red and delicate skin. Although the bright red coloring faded by P2 or P3, LPD pups remained more reddish in appearance than control pups, which transitioned from red to pink and then to a pale color with thicker skin and a developing hair coat.

Control pups developed a thin but consistent hair cover over their bodies by P6, achieving full hair coverage by P9-10. In contrast, F1 LPD offspring showed a 2–3-day delay in acquiring body hair, with significant hair growth not occurring until P8-9. Additionally, control pups could be sexed by P8, as female mice developed prominent nipples and genital dimorphism became apparent. LPD offspring, however, could not be consistently sexed until P11-12. By P20 F1 pups from the F0 fed an LPD presented with underdeveloped hair coats and decreased body dimensions (Figures D and E) when compared to controls at matched age (Figure F). These delayed growth patterns observed in F1 LPD offspring pups were consistent across different litters. Morphometric analysis of P20 F1 offspring revealed significant effects of LPD on development. Body weight (P20 NPD F1 = 16.3 ± 1.57g, n = 18; LPD F1 14.0 ± 1.57g, n = 17, p = 9e-4, Wilcoxon test) and kidney weight (P20 NPD 0.232 ± 0.0369g; LPD 0.179 ± 0.0297g, p = 0.00012, Wilcoxon test) were significantly reduced in LPD offspring (Fig. 2G and H). Notably, at P20, The KW/BW ratio was decreased in the LPD offspring (KW/BW NPD F1 = 0.0138 ± 0.00180g, 18; LPD F1 = 0.0127 ± 0.00133g, n = 17, p = 0.0048, Wilcoxon test) (Fig. 2I). LPD impacted body weight and kidney weight in both males and females to a similar extent, but statistical significance was not reached for males at LPD (Fig. 2J and K). Furthermore, glomerular counts at P20 were reduced in the F1 generation from F0 fed an LPD diet (NPD F1 = 158 ± 18, 37; LPD = 129 ± 21, 27, p = 0.00025, Wilcoxon test) (Fig. 2L). Morphological analysis of kidney sections at P20 illustrates how parental diet affects kidney development in offspring. The F1 P20 kidney section from the parental NPD appears more densely packed with cells, higher glomerular counts (black arrows), and less interstitial space, whereas the F1 P20 kidney section from the parental LPD group shows sparse glomeruli (Fig. 2M and N). Of note, at P20 glomeruli from LPD-fed offspring appear to be more variable in size (Fig. 2M and N).

These results highlight the continued adverse effects of prenatal protein restriction into early postnatal development, underscoring the critical window during which adequate nutrition is essential for optimal growth and organogenesis.

### 3.3 Long-term parental exposure to a low-protein diet impacts kidney development in adult animals

**Fig. 3:**
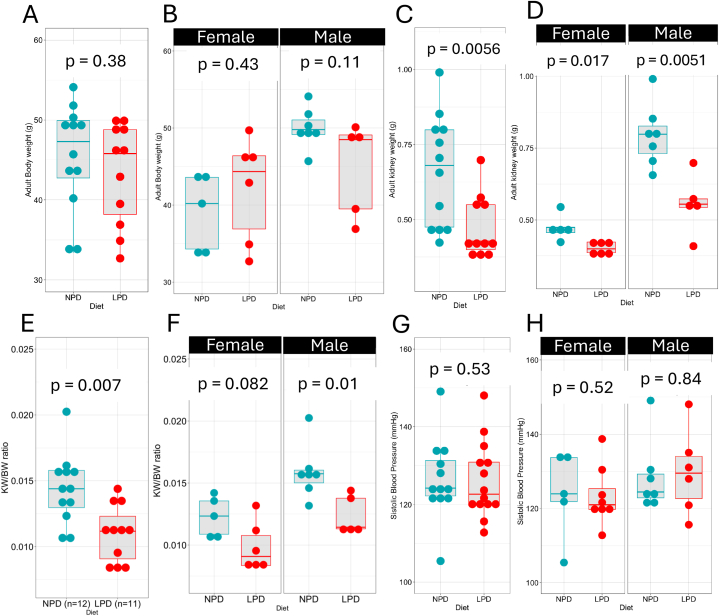
**LPD impact on mouse and kidney development persists in adult animals.** Body weight is not different in LPD vs NPD offspring at 3 months (BW mean ± sd NPD = 45.4 ± 6.67g; LPD = 43.3 ± 6.35g) (A). Body weight differences were normalized in adult males and females (B). The kidney weight remained reduced in adult animals (KW mean ± sd NPD = 0.661 ± 0.185g; LPD = 0.473 ± 0.104g) (C). The KW reduction was observed in both males and females (D). The KW/BW ratio remained decreased in the adult LPD offspring (KW/BW mean ± sd NPD = 0.0144 ± 0.00265; LPD = 0.0110 ± 0.00218g) (E). The reduction in the KW/BW was noticeable in both sexes, but statistical significance was only reached in males (F). There was no difference in systolic blood pressure measurements of F1 offspring from F0 parents fed an LPD or NPD at 3 months of age (NPD BP = 125 ± 10.3 mmHg; LPD BP = 126 ± 9.71 mmHg) (G, H). Sample size and p-value are reported in the figure. Values are presented as mean ± sd.

The impact of LPD on kidney development persists into adulthood (Fig. 3). At three months, there was a decreasing trend in body weight but no significant difference between LPD and NPD offspring (BW NPD F1 = 45.4 ± 6.67g, n = 12; LPD F1 = 43.3 ± 6.35g, n = 11, p = 0.38, Wilcoxon test) (Fig. 3A) in both males and females (Fig. 3B). However, kidney weight remained reduced in adult LPD animals (KW NPD F1 = 0.661 ± 0.185g; LPD F1 = 0.473 ± 0.104g, p = 0.0056, Wilcoxon test) (Fig. 3C), and this reduction was observed in both sexes. Interestingly, adult kidney weight was higher in males, regardless of the diet type (Fig. 3D). The KW/BW ratio remained decreased in adult LPD offspring (KW/BW NPD F1 = 0.0144 ± 0.00265g; LPD F1 = 0.0110 ± 0.00218g, p = 0.007, Wilcoxon test) (Fig. 3E), with statistical significance reached in males, but not females (Fig. 3F). No significant differences in systolic blood pressure were observed between NPD F1 and LPD F1 offspring at three months of age (NPD F1 BP = 125 ± 10.3 mmHg, n = 12; LPD F1 BP = 126 ± 9.71 mmHg, n = 14, p = 0.58, Wilcoxon test), in both sexes (Fig. 3G and H).

These findings indicate that the effects of prenatal LPD persist into adulthood, particularly affecting kidney size, which may have long-term implications for renal health and overall metabolic regulation.

### 3.4 Paternal contribution to LPD impact on kidney development

**Fig. 4:**
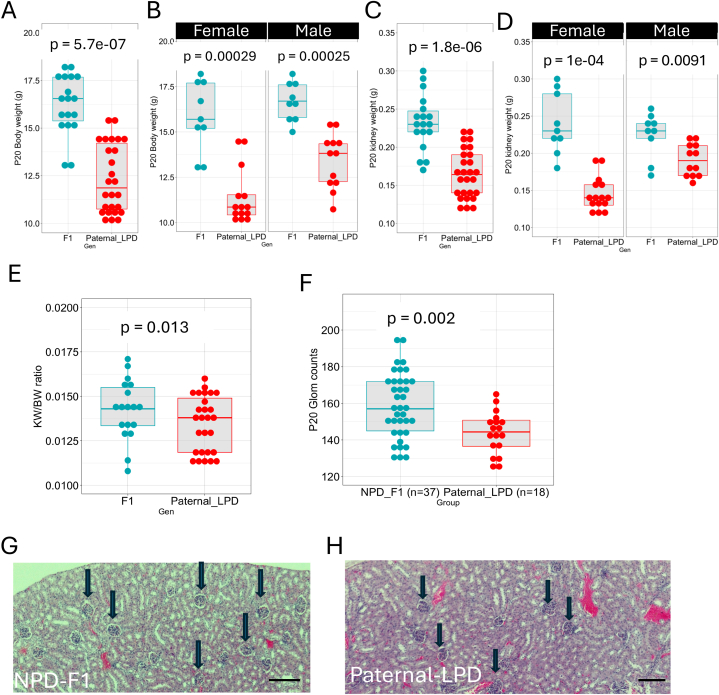
**Paternal contribution to LPD impact on mouse and kidney development.** Morphological analysis of P20 F1 offspring shows the impact of paternal LPD on normal kidney development. Paternal LPD leads to a reduction in body weight at P20 F1 offspring (F1 control BW = 16.3 ± 1.57g, n = 18; paternal LPD 12.2 ± 1.83g, n = 27, p = 5.7e-7, Wilcoxon test) in both sexes (A and B) and kidney weight (F1 control KW = 0.232 ± 0.0369g; paternal LPD 0.165 ± 0.0319g, p = 1.8e-6, Wilcoxon test) (C and D). KW/BW ratio was decreased (KW/BW F1 control = 0.0142 ± 0.00168g; paternal LPD = 0.0134 ± 0.00156g, p = 1.8e-6) (E). The glomerular counts at P20 were reduced in the F1 generation from the paternal-fed LPD (F1 = 158 ± 18; paternal LPD = 144 ± 11.6, n = 18, p = 0.002) (F). H&E staining of tissue sections from P20 kidney of paternal NPD and LPD groups. the round structures indicated with black arrows are glomeruli (G and H). Values are presented as mean ± sd. Scale bar 200 μm.

It is well-accepted that the maternal diet during the gestation and lactation periods affects the offspring's health [16,30,[41], [42], [43]]. However, a growing body of evidence suggests that the paternal diet also influences the offspring's normal development [44,45]. Previous research investigated the effects of paternal LPD on cardiovascular and metabolic functions in mice [46], however, the effects of paternal LPD on nephron endowment have not been studied in mice. For this reason, we decided to investigate the effects of paternal diet on kidney development in mice. The paternal contribution to the impact of LPD on kidney development is shown in Fig. 4. Morphological analysis of P20 F1 offspring showed that paternal LPD resulted in reduced body weight (F1 control BW = 16.3 ± 1.57g, n = 18; paternal LPD 12.2 ± 1.83g, n = 27, p = 5.7e-7, Wilcoxon test) in both sexes (Fig. 4A and B). Moreover, the kidney weight was also decreased in both sexes (F1 control KW = 0.232 ± 0.0369g; paternal LPD 0.165 ± 0.0319g, p = 1.8e-6, Wilcoxon test) (Fig. 4C and D). The KW/BW ratio was decreased, but it did not reach statistical significance (KW/BW F1 control = 0.0142 ± 0.00168g; paternal LPD = 0.0134 ± 0.00156g, p = 0.13) (Fig. 4E). More importantly, the glomerular counts at P20 were reduced in the F1 generation from the paternal-fed LPD (F1 = 158 ± 18, n = 37; paternal LPD = 144 ± 11.6, n = 18, p = 0.002) (Fig. 4F). Of note, with paternal LPD, the interquartile range appeared to be wider in almost all measurements, indicating a greater variability of the sample. Morphological analysis of kidney sections from the NPD Group shows that glomeruli are more frequent and more uniform, whereas sections from the paternal LPD show that the glomeruli are more sparse and more variable in size (Fig. 4G and H).

This evidence suggests that paternal diet also plays a significant role in determining offspring health outcomes, indicating that both maternal and paternal nutrition are critical factors in early development.

### 3.5 Transgenerational impact on F2 kidney development

**Fig. 5:**
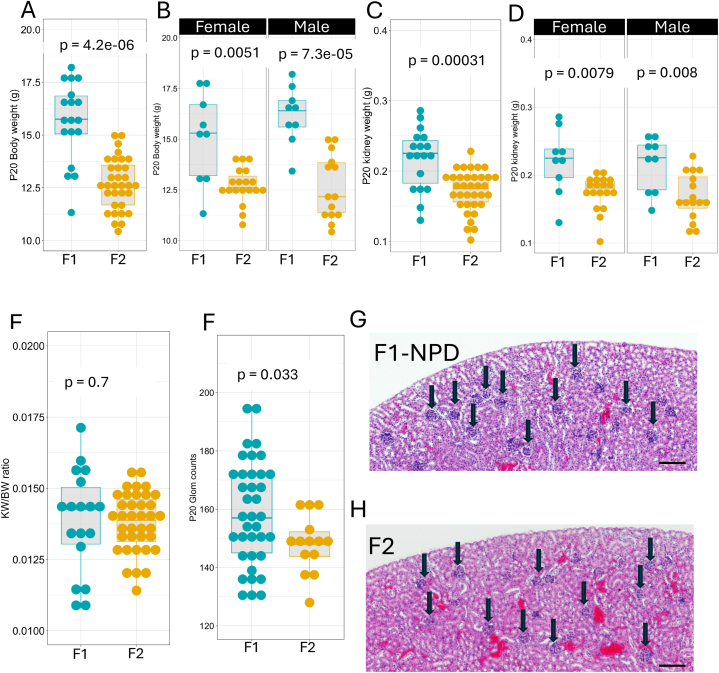
**Transgenerational impact of LPD on F2 kidney development.** P20 F2 offspring from F0 fed with an LPD shows reduced Body weight (NPD-F1 BW = 16.3 ± 1.57g, n = 18; F2 = 12.1 ± 1.85g, n = 31, p = 4.2e-8, Wilcoxon test) in both sexes (A and B). Kidney weight (KW F1 = 0.218 ± 0.0431g; F2 KW = 0.186 ± 0.116g, p = 0.0003, Wilcoxon test) was significantly reduced in LPD offspring in both sexes (C, D). The KW/BW ratio was decreased in the P20 LPD offspring (KW/BW F1 control = 0.0142 ± 0.00168g; F2 = 0.0153 ± 0.00905g, p = 0.7, Wilcoxon test) (E). The glomerular counts at P20 were reduced in the F1 generation from F0 fed an LPD (F1 = 158 ± 18, n = 37; F2 = 147 ± 9.8, n = 14, p = 0.033, Wilcoxon test) (F). H&E staining of tissue sections from P20 kidney of parental NPD and F2 from F0-fed LPD groups, the round structures indicated with black arrows are glomeruli (G and H). Values are presented as mean ± sd. Scale bar 200 μm.

A substantial body of evidence shows that an abnormal parental diet can have a transgenerational impact on offspring both humans and rats [16,35,45,[47], [48], [49]]. Thus, we sought to investigate whether, in mice, parental LPD would impact kidney development in multiple generations. To this end, F1 offspring from parents who received an LPD were weaned at P20 and put on NPD until five weeks of age. At five weeks of age, these mice were bred to produce an F2 generation (Fig. 1). P20 F2 offspring from F0 fed with LPD showed reduced body weight (F1 control BW = 16.3 ± 1.57g, n = 18; F2 = 12.1 ± 1.85g, n = 31, p = 4.2e-8, Wilcoxon test) in both sexes (Fig. 5A and B). Kidney weight was significantly reduced in LPD offspring in both sexes (KW F1 = 0.218 ± 0.0431g; F2 KW = 0.186 ± 0.116g, p = 0.0003, Wilcoxon test) (Fig. 5C and D). interestingly, the KW/BW ratio was decreased in P20 LPD offspring but did not reach statistical significance (KW/BW F1 control = 0.0142 ± 0.00168g; F2 = 0.0153 ± 0.00905g, p = 0.7, Wilcoxon test) (Fig. 5E). Glomerular counts at P20 were reduced in the F2 generation from F0 fed an LPD (F1 = 158 ± 18, n = 37; F2 = 147 ± 9.8, n = 14, p = 0.033, Wilcoxon test) (Fig. 5F). Morphological analysis of these specimens shows that glomeruli are more densely packed in the NPD group, while sections from the F2 from F0-fed LPD show fewer glomeruli (Fig. 5G and H).

These findings demonstrate that the adverse effects of prenatal LPD are not limited to the directly exposed generation but persist into subsequent generations.

### 3.6 Transgenerational impact on F3 and F4 kidney development

**Fig. 6:**
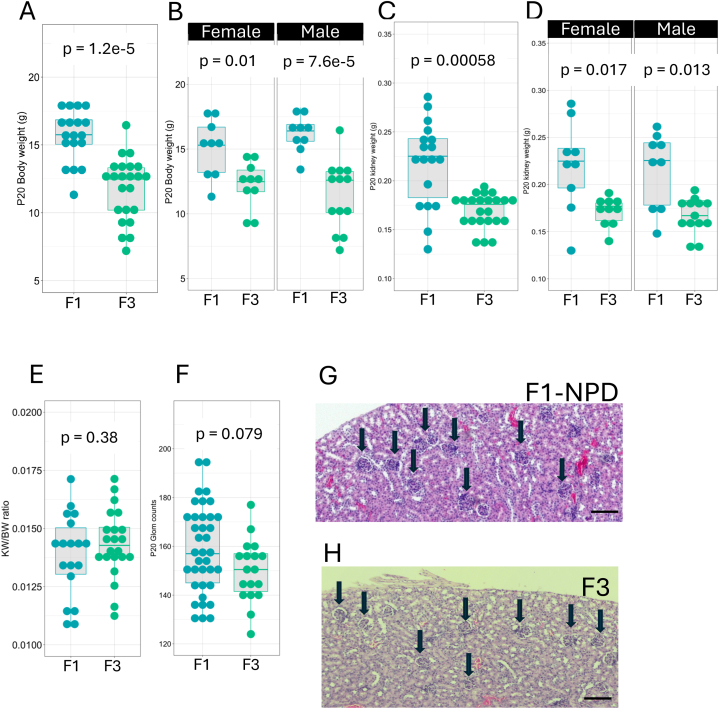
**Transgenerational impact of LPD on F3 kidney development.** P20 F3 offspring from F0 fed with an LPD shows reduced Body weight (F1 BW = 15.6 ± 1.90g, n = 18; F3 = 11.8 ± 2.32g, n = 23, p = 1.2e-5, Wilcoxon test) in both sexes (A and B). Kidney weight (F1 KW = 0.218 ± 0.0431g; F3 KW = 0.169 ± 0.0172g, p = 0.00058) was significantly reduced in LPD offspring in both sexes (C, D). The KW/BW ratio was decreased in the P20 LPD offspring (KW/BW F1 control = 0.0142 ± 0.00168g; F3 = 0.0148 ± 0.00259g, p = 0.38, Wilcoxon test) (E). The glomerular counts at P20 were reduced in the F1 generation from F0 fed an LPD (F1 = 159 ± 18, n = 37; F3 = 148 ± 15.3, n = 19, p = 0.079, Wilcoxon test) (F). H&E staining of tissue sections from P20 kidney of parental NPD and F2 from F0-fed LPD groups, the round structures indicated with black arrows are glomeruli (G and H). Values are presented as mean ± sd. Scale bar 200 μm.

Next, we sought to determine whether F0 LPD would still impact nephron endowment on an F3 generation and F4. Fig. 6 shows the transgenerational impact of LPD on F3 kidney development. P20 F3 offspring from F0 fed with LPD showed reduced body weight (F1 BW = 15.6 ± 1.90g, n = 18; F3 = 11.8 ± 2.32g, n = 23, p = 1.2e-5, Wilcoxon test) in both sexes (Fig. 6A and B). Kidney weight was significantly reduced in LPD offspring in both sexes (F1 KW = 0.218 ± 0.0431g; F3 KW = 0.169 ± 0.0172g, p = 0.00058) (Fig. 6C and D). As observed for F2, we did not detect KW/BW ratio differences in F3 P20 LPD offspring compared to F1 generation from F0 parents on NPD (KW/BW F1 control = 0.0142 ± 0.00168g; F3 = 0.0148 ± 0.00259g, p = 0.38, Wilcoxon test) (Fig. 6E). Interestingly, the average glomerular counts at P20 were reduced in the F3 generation from F0 fed an LPD. But this reduction was not statistically significant (F1 = 159 ± 18, n = 37; F3 = 148 ± 15.3, n = 19, p = 0.079, Wilcoxon test) (Fig. 6F). H&E-stained section of these groups indicates that glomeruli densities are normalized in F3 compared to controls (Fig. 6G and H).

**Fig. 7:**
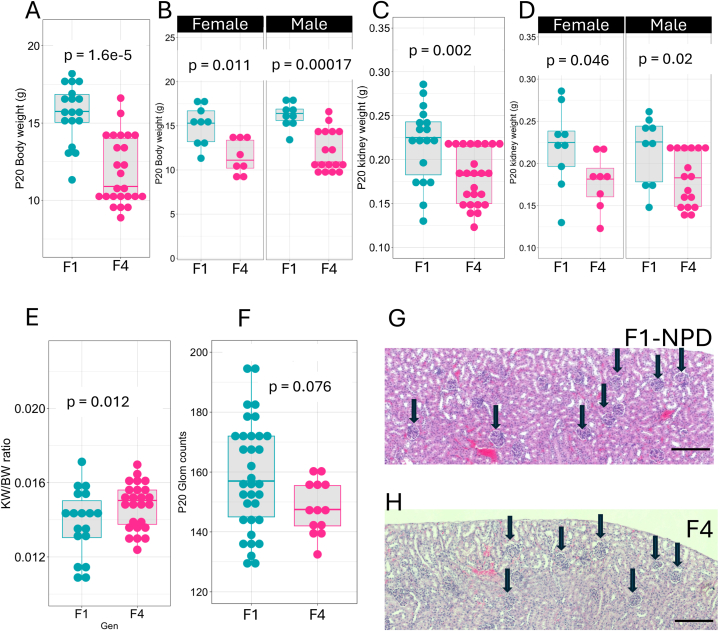
**Transgenerational impact of LPD on F4 kidney development.** P20 F4 offspring from F0 fed with an LPD shows reduced Body weight (F1 BW = 15.6 ± 1.90g, n = 18; F4 = 11.9 ± 2.58g, n = 25, p = 1.6e-5, Wilcoxon test in both sexes (A and B). Kidney weight (F1 KW = 0.218 ± 0.0431g; F4 KW = 0.180 ± 0.0311g, p = 0.002) was significantly reduced in LPD offspring in both sexes (C, D). The KW/BW ratio was decreased in the P20 LPD offspring (KW/BW F1 = 0.0138 ± 0.00180g; F4 = 0.0152 ± 0.00156g, p = 0.12, Wilcoxon test) (E). The glomerular counts at P20 were reduced in the F1 generation from F0 fed an LPD (F1 = 158 ± 18, n = 37; F4 = 148 ± 8.83, n = 13, p = 0.076, Wilcoxon test) (F). H&E staining of tissue sections from P20 kidney of parental NPD and F2 from F0-fed LPD groups, the round structures indicated with black arrows are glomeruli (G and H). Values are presented as mean ± sd. Scale bar 200 μm.

Although the differences in glomerular counts were not significant in the F3 generation, we noticed that body weight and kidney weight were still impacted. Thus, we sought to study the F4 generation. Fig. 7 shows the transgenerational impact of LPD-fed F0 on F4 kidney development. At P20, the F4 offspring from F0 fed with LPD remained with reduced body weight (F1 BW = 15.6 ± 1.90g, n = 18; F4 = 11.9 ± 2.58g, n = 25, p = 1.6e-5, Wilcoxon test). This phenomenon was observed in both sexes (Fig. 7A and B). Interestingly, kidney weight was also impacted (F1 KW = 0.218 ± 0.0431g; F4 KW = 0.180 ± 0.0311g, p = 0.002) (Fig. 7C and D). We did not observe differences in KW/BW in the F4 group (KW/BW F1 = 0.0138 ± 0.00180g; F4 = 0.0152 ± 0.00156g, p = 0.12, Wilcoxon test) (Fig. 7E). There were no significant differences in glomerular counting between the F1 and F4 generations (F1 = 158 ± 18, n = 37; F4 = 148 ± 8.83, n = 13, p = 0.076, Wilcoxon test) (Fig. 7F). As for F3, the H&E-stained section of F4 from F0 fed an LPD shows that glomeruli densities are normalized when compared with F1 from an NPD-fed F0 generation compared to controls (Fig. 7G and H).

## 4 Discussion

In this study, we demonstrated that protein restriction impacts kidney development across multiple generations. We also demonstrated that a low-protein diet in either both parents (male and female) or the father alone significantly reduced nephron endowment in mouse offspring. Mice born following protein-restricted gestation exhibited low birth weight and smaller kidneys. At birth, they showed oligonephropathy, which persisted later in life (see Fig. 2, Fig. 3). We observed a similar reduction in kidney weight and nephron endowment when a male fed an LPD was bred with a female on NPD (Fig. 4). This suggests a significant contribution from the father to the final nephron endowment.

Despite the clinical relevance, there is limited data on the transgenerational impact of a low-protein diet on kidney development, particularly in mice. Most previous research, used rats as a model organism and focused on the impact of a gestational (maternal-only) low-protein diet on kidney development and programming of hypertension later in life [[50], [51], [52], [53], [54]]. This bias towards rats may stem from early studies demonstrating that a maternal low-protein diet significantly impacts kidney development [[55], [56], [57], [58]]. Because mice are highly valuable for genetic manipulation and mechanistic analyses, we believe that studying the transgenerational impact of diet on offspring development in mice is important for advancing scientific understanding of the developmental origin of adult diseases and uncovering species-specific mechanisms of resilience to environmental stress.

A recent study shows that a low-protein diet during pregnancy leads to different effects on blood pressure programming in male and female mice, suggesting that sex may be an important biological variable regarding the developmental origin of hypertension. The study found that both moderate and severe protein restriction during pregnancy had varying impacts on male and female offspring [59]. The study did not report counts of nephrons in the offspring of parents fed an LPD, failing to address an important risk factor for the development of hypertension and chronic kidney disease later in life. Our study highlights the significant impact of parental diet on the embryonic programming of hypertension by measuring nephron endowment in the offspring of LPD-fed parents. Additionally, our findings suggest that poor parental nutrition influences not just the first generation but extends its effects to multiple generations. A similar pattern has been observed in humans whose parents or grandparents experienced starvation, with the second and third generations still showing the impact of these food restrictions [16,[60], [61], [62], [63]].

Our results show that, at birth, body weight and kidney weight remained reduced for four generations, however, the nephron counts were normalized in the F3 and F4 generations (Fig. 6, Fig. 7). Interestingly, F3 and F4 showed variable penetrance and even phenotype expressivity. This observed variability might have resulted from several factors. For instance, normalized glomeruli counts in F3 and F4 might have occurred due to epigenetic resetting. Epigenetic changes induced by dietary factors, such as a low-protein diet in the F0 generation, could be reset over subsequent generations (F3 and F4). This resetting can occur during germline reprogramming, which could explain why the effects diminish in F3 and F4. Studies show that nutritional interventions can induce epigenetic changes, but these effects might or might not persist indefinitely across generations due to reprogramming during early embryonic development [[64], [65], [66], [67], [68], [69]].

Moreover, F3 and F4 may have developed compensatory mechanisms that help restore normal glomerular counts. For example, previous studies have shown that low-protein diets can trigger adaptative or maladaptive responses to restore homeostasis, such as alterations in energy intake and metabolic pathways, or mechanisms of blood pressure regulation which might reduce the observable effects in later generations [47,70]. The mechanisms responsible for transgenerational effects in developmental programming are still poorly understood.

Finally, the observed variability in glomerular counts of F3 and F4 generations may have resulted from other factors, such as threshold effects and sample size limitations. The p-values for F3 and F4 (p = 0.079 and p = 0.076) suggest that the differences may not have been large enough to reach statistical significance. This might reflect a threshold effect, where changes were present but not strong enough to be detectable due to the large biological variability observed in CD1 outbred strains.

The effects of maternal LPD on rat offspring development and blood pressure have been investigated before [50,51,71,72]. However, previous studies were limited to a single generation and mostly focused on maternal protein restriction. The transgenerational effects of protein restriction on nephron mass in mice remain poorly explored. Maternal and paternal starvation in humans is correlated with obesity and diabetes in the descendants, as well as with hypertension and chronic kidney disease later in life [14,16,19,21,58,[60], [61], [62], [63],[73], [74], [75], [76], [77]]. Corroborating with these observations in humans, our study found that protein restriction in the F0 generation led to abnormal kidney development across multiple generations. These results highlight the profound and lasting impact of prenatal nutrition on renal health and underscore the importance of adequate.

There is an established relationship between nephron counts and hypertension in both humans and rodents [14,20,[78], [79], [80], [81], [82]]. Furthermore, previously published data on rats demonstrated that parental LPD resulted in oligonephropathy at birth and increased blood pressure later in life [47]. In our cohorts, however, we did not observe differences in systolic blood pressure between the offspring of different diet groups at 12 weeks of age (NPD systolic BP = 125 ± 10.3 mmHg; LPD systolic BP = 126 ± 9.71 mmHg, p = 0.53), which is consistent with previous measurements, in mice, showing that blood pressure increases becomes significant after 18 weeks of age in the offspring of paternal LPD [46]. We did observe a higher variability (full range and interquartile range) in systolic blood pressure in the offspring of the parental LPD group compared to the offspring of parental NPD. This variability was higher than that observed for rats in previous publications [47,50]. Likely, these differences resulted from the distinction between species, sample size, age of animals, and equipment sensitivity. For future studies, it would be interesting to follow variations in blood pressure in older animals and sensitivity to challenges such as a high-salt diet, high-fat diet, and or angiotensin stimulation.

An interesting hypothesis to explain the lack of difference in systolic blood pressure between NPD and LPD F1 offspring at three months of age could be the activation of compensatory mechanisms or the occurrence of an age-dependent event. Other studies have reported sexually dimorphic programming of hypertension in mice, with measurable changes in blood pressure emerging only after 24 weeks of age [78,79]. Because congenital oligonephropathy leads to progressive kidney damage [13], perhaps the offspring of the LPD group either would show hypertension at later time points or the appearance of hypertension may depend on a second hit, such as exposing the LPD offspring to a high-fat diet, as observed with rats [47]. Our thoughts are that the age-dependent decline in kidney function will lead to the development of hypertension in the offspring of LPD-fed parents, regardless of any compensatory mechanism.

Recently, greater attention has been devoted to the importance of paternal diet to offspring development [33,44,45]. Morgan et al. reported that a paternal LPD impacts normal inter-generational metabolic homeostasis leading to hepatic and inflammatory alterations in mice [45].

To test whether paternal LPD could impact nephron endowment in mice, we exposed the father, but not the mother, to an LPD, and we observed a significant paternal component orchestrating nephron endowment in the developing kidney. Although other studies have reported several metabolic changes in the offspring of paternal malnutrition [33,44,45]. Our study is likely the first one to report the impact of paternal LPD on offspring nephron mass endowment. Our results, to some extent, recapitulate observations in humans who faced a deficient diet pre-conceptually or during gestation, resulting in a higher risk for hypertension, metabolic diseases, and the development of chronic kidney disease [17,31,35,36,41,80,81]. Moreover, our study shows that not only the mother, but the father's nutritional status is also important for proper kidney development.

It is estimated that 15–20 % of all births worldwide, about 20 million newborns annually, are low birth weight infants [82]. The definition of LBW in humans was infants at the 10th percentile of body weight. All mouse pups were weighed at birth and the 10th percentile for control mice was calculated to be 1.42g. Interestingly, the P0 mean body weight from the NPD group was 1.61 ± 0.15g while the LPD mean body weight was 0.87 ± 0.17g, nearly half the control group. Notably, kidney weight was also lower in the LPD offspring across multiple generations. At P20 the LPD F1 offspring looked stunt and underdeveloped compared to controls (Fig. 2D–F).

This significant reduction in body and kidney weight among LPD offspring highlights the profound impact of maternal protein restriction on fetal growth. Utilizing this model system to screen for potential compounds that can mitigate the effects of an LPD on embryo and kidney development is of great interest. Previous studies have identified compounds in deficient diets that result in faulty kidney development. Interestingly, interventions with supplementation of specific compounds rescued the low nephron endowment phenotype [4,[83], [84], [85], [86], [87]].

Identifying key metabolites necessary for proper kidney organogenesis could lead to the development of supplements that ensure proper fetal and kidney development, particularly in impoverished communities or with people whose predecessor generation had faced food scarcity [16,31,48,49,80].

Given that the murine kidney finishes development after birth, future studies shall explore the roles of lactation kidney development, perhaps by cross-fostering pups from LPD to NPD dams to measure the impact of normal lactation on kidney development, and whether it would impact future generations. Normal lactation has been shown to restore nephron endowment, preventing hypertension in rats whose development was hampered by placental restriction [88].

The findings of our study help elucidate the long-term impacts of maternal and paternal diet on offspring health, which is crucial for understanding potential interventions to mitigate adverse outcomes. Our results provide compelling evidence for the profound and lasting impact of maternal and paternal nutrition on offspring development. The significant reduction in body weight and kidney size in LPD offspring across multiple generations highlights the critical need for adequate prenatal nutrition. Future studies should focus on identifying specific compounds that can rescue the effects of an LPD on embryo and kidney development, paving the way for interventions that promote healthy fetal development and long-term health.

### 4.1 Limitations of the study

This study's use of tail-cuff BP measurements may have masked time-dependent changes in blood pressure. More precise methods, such as telemetry, would allow for more sensitive measurements of hemodynamic parameters in these mice. Additionally, visual counts of glomeruli on H&E sections may not capture small yet consistent reductions in nephron numbers across experimental groups. More accurate methods have been described and can increase the accuracy of total glomeruli per kidney across different generations [5,47].

The study did not evaluate markers of CKD such as glomerulosclerosis, interstitial fibrosis, proteinuria, and eGFR, which could provide further insights into the long-term impact of LPD on kidney function.

## CRediT authorship contribution statement

**Fabiola Diniz:** Project administration, Methodology, Investigation. **Francesca Edgington-Giordano:** Methodology, Investigation. **Nguyen Yen Nhi Ngo:** Investigation. **Gal Caspi:** Investigation. **Samir S. El-Dahr:** Writing – original draft, Funding acquisition, Data curation. **Giovane G. Tortelote:** Writing – review & editing, Writing – original draft, Supervision, Resources, Project administration, Methodology, Investigation, Funding acquisition, Formal analysis, Data curation, Conceptualization.

## Declaration of competing interest

The authors declare the following financial interests/personal relationships which may be considered as potential competing interests:

Samir El-Dahr reports financial support was provided by 10.13039/100000002National Institutes of Health. If there are other authors, they declare that they have no known competing financial interests or personal relationships that could have appeared to influence the work reported in this paper.
